# SSR markers associated to early leaf spot disease resistance through selective genotyping and single marker analysis in groundnut (*Arachis hypogaea* L.)

**DOI:** 10.1016/j.btre.2017.07.005

**Published:** 2017-08-10

**Authors:** Adama Zongo, Pawan Khera, Mahamadou Sawadogo, Yaduru Shasidhar, Manda Sriswathi, Manish K. Vishwakarma, Philippe Sankara, Bonny R. Ntare, Rajeev K. Varshney, Manish K. Pandey, Haile Desmae

**Affiliations:** a International Crops Research Institute for the Semi-Arid Tropics (ICRISAT), BP 320 Bamako, Mali; b International Crops Research Institute for the Semi-AridTropics (ICRISAT), Hyderabad 502 324, India; c University Ouaga 1Joseph Ki Zerbo, BP 7021 Ouagadougou, Burkina Faso

**Keywords:** Groundnut, Early leaf spot, Simple sequence repeat, Single marker analysis, Selective genotyping

## Abstract

Groundnut (*Arachis hypogaea* L.) is an important oilseed and food crop of the world. Breeding for disease resistance is one of major objectives in groundnut breeding. Early leaf spot (ELS) is one of the major destructive diseases worldwide and in West Africa, particularly in Burkina Faso causing significant yield losses. Conventional breeding approaches have been employed to develop improved varieties resistant to ELS. Molecular dissection of resistance traits using QTL analysis can improve the efficiency of resistance breeding. In the present study, an ELS susceptible genotype QH243C and an ELS resistant genotype NAMA were crossed and the F_2_ population genotypic and F3 progenies phenotypic data were used for marker-trait association analysis. Parents were surveyed with 179 simple sequence repeat (SSR) markers out of which 103 SSR markers were found to be polymorphic between the parents. These polymorphic markers were utilized to genotype the F_2_ population followed by marker-trait analysis through single marker analysis (SMA) and selective genotyping of the population using 23 resistant and 23 susceptible genotypes. The SMA revealed 13 markers while the selective genotyping method identified 8 markers associated with ELS resistance. Four markers (GM1911, GM1883, GM1000 and Seq13E09) were found common between the two trait mapping methods. These four markers could be employed in genomics-assisted breeding for selection of ELS resistant genotypes in groundnut breeding.

## Highlights

• Marker trait association for ELS in groundnut.
• Single marker analysis (SMA) and selective genotyping approaches identified markers associated with ELS resistance.
• Four markers were found common between the two trait mapping methods.

## 1 Introduction

Groundnut or peanut (*Arachis hypogaea* L.), originated in South America, is one of the most important oilseeds and food crops cultivated in the semi-arid tropics. The cultivated groundnut is tetraploid (2*n* = 4*x* = 40). It is member of genus *Arachis* and family Leguminosae [1]. The agro-morphological diversity within the crop, particularly the differences in the branching pattern and presence of reproductive node on the main stem, allowed to distinguish the two cultivated subspecies i.e. *A. hypogaea* subsp. *hypogaea* and *A. hypogaea* subsp. *fastigiata*. The subspecies are further divided into botanical varieties. The subspecies *hypogaea* is divided into *hypogaea* (virginia) and *hirsuta,* while the subspecies *fastigiata* into *fastigiata* (valencia), *vulgaris* (spanish), *peruviana* and *aequatoriana* [1].

In 2014, groundnut was grown in 115 countries covering a total area of about 26.54 million (M) hectares (ha) with a global production of about 43.91 M tons and an average yield of about 1655 kg/ha [2]. The Asian continent ranks first with over 58.3% of world production, followed by the African continent (31.6%), American continent (10.0%) and Oceania (0.1%). The major producing countries are China (16.55 M tons), India (6.56 M tons), Nigeria (3.41 M tons), USA (2.35 M tons) and Sudan with 1.77 M tons [2]. In Africa, groundnut production has grown significantly from year 1990 to 2000. This growth is mainly due to increased production in West African countries such as Nigeria, Senegal, Ghana, Burkina Faso and Mali [3]. For example Nigeria, the third largest producer in the world, accounted for about a fourth of groundnut production in Africa in 2014 [2].

Groundnut is a good source of fat, protein and minerals and hence it plays important role in human nutrition. Its seed contains 48–55% oil and 26–28% protein, and is a rich source of dietary fiber, minerals and vitamins [4]. The haulms and groundnut cake are important sources of animal feed. In addition, groundnut has ability to fix atmospheric nitrogen to the soil to help in the maintenance of soil fertility. The hardiness, plasticity, the multiplicity of uses of groundnut makes it one of the most useful legume crops.

Despite its importance, the productivity of groundnut is severely constrained by several biotic and abiotic factors. The yield of groundnut in Africa is very low, around 1 ton/ha, compared to global average yield of about 2 ton/ha. Among the major constraints, biotic factors particularly foliar diseases constitute a serious yield limiting challenge in groundnut production. Early leaf spot (ELS) caused by *Cercospora arachidicola* Hori and late leaf spot (LLS) caused by *Phaeoisariopsis personata* (Bert and Curtis) Deigton are the most destructive foliar fungal diseases [5]. Groundnut yield losses pertaining to these two diseases are estimated to reach up to 50–70% along with adverse effects on the quality of the produce [6].

In order to reduce the impact of these diseases, control methods include use of chemical and resistant varieties among others. The usage of fungicides allows good control, however majority of smallholder farmers cannot use them since they lack the financial resources and technical expertise required to use them [7]. Moreover, the use of fungicides is not a cost-effective approach for smallholder farmers. In addition, use of fungicides has negative effects on the environment as well as on human health. Genetic approach involving breeding for innate foliar disease resistance are considered sustainable and cost effective to reduce the impact of leaf spots. Studies were conducted to identify or develop resistant or tolerant varieties to these diseases through conventional breeding. The complex nature of inheritance with recessive genes conferring resistance has hindered the progress of disease resistance breeding [8].

The breeding efficiency for disease resistance can be enhanced by employing new biotechnological tools such as use of DNA markers for mapping and tagging of the markers with desirable traits [8], [9], [10]. Several studies have demonstrated that molecular technology assisted breeding has significant advantages than conventional breeding particularly for traits which are difficult to manage through phenotypic selection [11], [12]. Among the molecular markers, microsatellites or simple sequence repeats (SSRs) have received extensive attentions owning to their advantages of high reproducibility, co-dominant inheritance and high information content [13]. Constructing a molecular linkage map is now routine to trace the valuable alleles in a segregating population. Mapping population plays a crucial role in linkage map construction. Genetically diverse parents are selected for developing a mapping population to generate complete linkage map with large number of molecular markers.

Selective genotyping offers an alternative resourceful approach for deciphering trait linked markers, in which DNA markers are assayed only on the most genetically informative progeny. Hence those with extremely high and/or low phenotypic values for a trait of interest are only subjected to the marker-trait analysis. This allocation of genotyping resources only to selected progeny can reduce genotyping costs with little loss of information, and/or for validation and fine-mapping of QTL that have been detected. This concept was introduced by Lebowitz et al. [14], who used the term ‘trait-based analysis’ to refer to approaches to QTL mapping in which marker allele frequencies are compared between groups of progeny selected based on trait values. Lander and Botstein [15] introduced the more general term ‘selective genotyping’ for QTL mapping based on selected groups of progeny, and suggested that QTL analysis in this case could also be based on the usual marker-based approaches that compare phenotypic values among marker genotype classes.

Sun et al. [16] indicated that QTL mapping based on selective genotyping is more powerful than simple interval mapping method but less powerful than composite interval mapping method. Lebowitz et al. [14] and Gallais et al. [17] have discussed the theory and experimental design for analysis of marker allele frequencies in classes of progeny defined on the basis of quantitative trait values. Both authors concluded that trait-based analysis of selectively genotyped progeny can be a useful alternative to marker-based analysis of all individuals in a population, when only one quantitative trait is of interest. Xu et al. [18] have also concluded from simulation analyses that selective genotyping can be used to replace the entire population genotyping approach.

The present study was conducted to identify SSR markers associated to ELS disease resistance through selective genotyping.

## 2 Materials and methods

### 2.1 Mapping population

The F_2_ mapping population comprising of 82 F_2:3_ lines developed from the cross QH243C × NAMA was used for this study. QH243C belongs to Spanish bunch and is a high yielding cultivar in Burkina Faso; however it is susceptible to ELS. The genotype NAMA belongs to Virginia bunch and is highly resistant to ELS. The mapping population was developed at ICRISAT Mali. The F_2_ and F_3_ progenies were used for genotyping and phenotyping, respectively. The field experiment for phenotyping was carried out at ICRISAT Mali research station while the genotyping was done at ICRISAT Patancheru, India.

### 2.2 Phenotyping for early leaf spot disease

A set of 82 F_2_ individual plant and 46 F_3_ mapping population (23 resistant and 23 susceptible) along with the parental genotypes was phenotyped for ELS disease resistance. Phenotyping of mapping population was done during 2013 rainy season for F_2_ population and 2014 rainy season for F_3_ mapping population at ICRISAT Mali station under natural infestation. This station has been known to be a hotspot for ELS. The 23 resistant and 23 susceptible genotypes were obtained from F_2_ individual plant phenotyping. Seed of each F_3_ progeny was planted in a 4 m row spaced at 50 cm, and intra row spacing was 15 cm. Randomized complete block design with 3 replications was used to raise the F_3_ population. The seeds were treated with the fungicide APRON STAR 42W before sowing. Disease scoring for ELS was done at 40 days (ELS_I), 60 days (ELS_II) and 80 days (ELS_III) after sowing, by using a modified 9-point scale [19]. Disease score of 1 was given if there was 0% infection; 2 for 1–5%; 3 for 6–10%; 4 for 11–20%, 5 for 21–30%; 6 for 31–40%; 7 for 41–60%, 8 for 61–80% and 9 for 81–100% infection were recorded. Plants with a disease score of 1–3, 4–6 and 7–9 were designated as being resistant, moderately resistant and susceptible, respectively [20].

### 2.3 DNA extraction and genotyping with SSR markers

Firstly, young leaf tissues of the F2 plants were sampled and kept in a freezer at −80 °C. Then, for genotyping, only DNA of extreme progenies (i.e., 23 resistant and 23 susceptible) along with two parental lines were subsequently used. DNA was extracted using modified cetyltrimethyl ammonium bromide (CTAB) extraction method [21]. DNA quality and quantity were checked on 0.8% agarose gels and DNA concentration was normalized to get 5 ng/μl for further genotyping work.

Initially the parents QH243C and NAMA were screened for polymorphism by using 179 available SSR markers [21], [22], [23], [24], [25], [26]. One hundred three (103) markers were found to be polymorphic between the parents QH243C and NAMA. Based on the phenotyping data, the 46 F_2_ lines were selected for genotyping with the103 polymorphic SSR markers.

Polymerase chain reactions (PCR) were performed as described by Varshney et al.[27] with some modifications. The final reaction volume was 7 μl. The recipes for PCR reaction mixture for all the labeled and unlabelled primers were common except the volume of sterile distilled water. The PCR reaction was prepared in 384-well plates containing 2 μl template DNA (5 ng), 0.7 μl of 10× Taq buffer containing MgCl_2_ (50 mM), 0.7 μl of dNTP (2 mM), 0.7 μl of primers (5pm/μl) (forward and reverse), 0.04 μl of Taq polymerase (Genei 5U/μl), 0.25 μl of dye (2pm/μl) and 2.41 μl, 2.66 μl of sterile double distilled water for unlabelled and labeled primers, respectively.

Touchdown PCR program was used to minimize spurious amplification [28]. The program is as follows: 94 °C for 3 min to allow samples to denature, followed by 5 cycles of 94 °C for 20 s, 65 °C for 20 s, and 72 °C for 30 s, the annealing temperature was decreased 1 °C per cycle in subsequent cycles until the temperature reached 52 °C or 56 °C for the different touchdown programs. Products were subsequently amplified for 40 cycles at 94 °C for 20 s, 56 °C for 20 s, and 72 °C for 30 s, followed by a final extension for 20 min. The PCR products were tested on 1.2% agarose gel to ensure the successful amplifications. After amplification, the PCR products were separated through capillary electrophoresis (ABI 3730). Allele sizing was carried out using the GeneMapper 4.0 software.

### 2.4 Statistical analysis

#### 2.4.1 Phenotypic data

The analysis of variance at different stages of ELS disease scoring was done using the software GenStat 15th edition. The analysis allowed testing the significance of difference between the genotypes. Phenotypic coefficient of variance (PCV), genotypic coefficient of variance (GCV) and heritability in broad sense (H^2^) were estimated for all stages of disease scoring.

#### 2.4.2 Single marker and selective genotyping analysis

Single marker analysis (SMA) was performed using the F_2_ genotypic data of 103 polymorphic markers and F_3_ phenotypic data of ELS disease score to identify potential SSR markers associated ELS resistance. Simple regression method was used with the following linear equation:

y = b0 + b1x + e

Where, y = phenotypic trait value; b0 = population mean; b1x = function of the molecular marker and e = error. The potential relationship between the marker and trait was established considering the significance of the regression coefficient at 5%, 1% and 0.1% probability. The phenotypic variance explained was expressed in terms of adjusted R^2^ values. The analysis was performed using QTL Cartographer software. Parents and progenies allelic contribution was estimated for resistant and susceptible group using Microsoft Office Excel software.

## 3 Results

### 3.1 Phenotyping of mapping population

Analysis of variance revealed significant differences between the mapping populations for ELS at all scoring stages. The phenotypic data of mapping population showed near normal distribution, but slightly skewed toward susceptibility at the last stage, i.e., ELS_III (80 days after sowing). The mean disease score of 2.93 for parent NAMA for ELS showed consistently lower disease incidence than QH243C (7.35) at all the scoring stages (Table 1).

**Table 1:** Mean of parents and mapping population and estimates of phenotypic coeefficient of variation (PCV) and genotypic coefficient of variation (GCV) and broad sense heritability (H^2^b.s) for ELS in QH243C x NAMA mapping population.

ELS Scoring stage	Mean			GCV	PCV	H^2^b.s
	QH243C	NAMA	F_2_ population			
ELS_I	2.33	2.00	2.33 ± 0.03	7.87	13.16	35.77
ELS_II	4.32	2.02	2.91 ± 0.074	19.03	23.19	67.36
ELS_III	7.35	2.93	5.04 ± 0.14	24.18	26.56	82.85

The estimated genetic parameters revealed from moderate to high PCV and from low to high GCV for ELS. The PCV was high at the last two stages of scoring (23.18 for ELS_II and 26.56 ELS_III) while moderate to high GCV (19.03–24.18%) was observed for the two scoring stages. The heritability ranged from 35.77 to 82.85%.

### 3.2 Marker analysis on parents and mapping populations

The susceptible and resistant parents (QH243C and NAMA) were surveyed with 179 SSR primers to identify polymorphic markers that would discriminate the two parents. All the 179 markers showed quality amplification in both genotype and 103 SSR markers (57.54%) showed polymorphism between the two parental lines. Genotyping data were obtained on the complete set of 46 F_2_ selected lines (23 resistant and 23 susceptible) for 103 markers.

### 3.3 Marker-trait analysis using single marker analysis

Simple linear regression was calculated using phenotypic traits and genotype of each marker. The results indicated that 13 markers were linked to ELS disease resistance out of the 103 markers (Table 2). The phenotypic variation explained (R^2^%) by these markers ranged from 3.18 (Seq19G07) to 23.25% (GM1878). Among the 13 markers linked to ELS disease resistance, markers Seq13E09, GM1883, GM2745, GM1878, TC6E01 and IPAHM509 were considered important as they accounted for more than 10% of phenotypic variation explained (R^2^%) of the trait. This indicates that these markers were associated with ELS disease as indicator for resistance genes.

**Table 2:** Single marker analysis for ELS in QH243C × NAMA mapping population.

Linkage group	Marker	F probability	R^2^%
A03	Seq19G07	0.025^a^	3.18
A04	GM2480	0.012^a^	5.86
A05	TC6E01	0.0001^c^	18.12
A06	IPAHM509	0.008^b^	11.67
A06	AH4-101	0.016^a^	3.86
A09	GM1878	0.001^c^	23.25
A09	GM1911	0.047^a^	5.31
A10	Seq3E10	0.014^a^	7.04
A10	GA131	0.001^c^	5.76
B03	Seq13E09	0.014^a^	10.68
B03	GM1883	0.003^b^	13.24
B07	GM2745	0.004^b^	16.77
B07	GM1000	0.042^a^	3.46

a significant at 5%.

b significant at 1%.

c significant at 0.1; R^2^%: phenotypic variation explained.

Surprisingly, the markers IPAHM509, Ah4-101, GA131 and GM2745 also showed an effect in ELS disease resistance through SMA but the majority of allele was contributed by the susceptible parent QH243C (Table 3). Their allelic contribution for the resistant bulk ranged from 3.2 to 30.4% for the resistant (NAMA) and 43.5 to 82.6% for susceptible parent (QH243C).

**Table 3:** Contribution of allele in resistant bulk for IPAHM509, Ah4-101, GA131 and GM2745 through single marker analysis.

Marker	Resistant Bulk		
	%A	%B	%H
IPAHM509a	4.3	82.6	4.3
AH4-101	3.2	69.6	13.0
GA131	4.3	43.5	30.4
GM2745	30.4	56.5	0.0

%A: allelic contribution of resistant parent (NAMA); %B: allelic contribution of susceptible parent (QH243C); %H: allelic contribution of hybrids

### 3.4 Selective genotyping for marker-trait association

All the 103 polymorphic markers were used for generating genotyping data following selective genotyping method. The genotyping data generated on 23 resistant and 23 susceptible F_2_ lines were used for identification of significantly associated markers. The analysis identified 8 linked markers (GM1911, GM1883, GM1000, Seq13A07, GM1988, GM2638, IPAHM245 and Seq13E09) for ELS (Table 4). Of these 8 markers, 4 markers − GM1911, GM1883, GM1000 and Seq13E09 – were common with SMA. Most of the alleles from resistant parent NAMA had a major effect on resistance with allelic contribution ranging from 17.4 to 65.2% and 4.3 to 17.4% for resistant and susceptible sets, respectively.

**Table 4:** Contribution of allele from resistant and susceptible set through selective genotyping.

Marker	Resistant Bulk (3.5 score)			Susceptible bulk (7.5 score)		
	%A	%B	%H	%A	%B	%H
GM1911	65.2	13.0	17.4	17.4	8.7	69.6
GM1883	60.9	0.0	34.8	17.4	4.3	65.2
GM1000	47.8	0.0	39.1	17.4	0.0	65.2
Seq13E09	17.4	56.5	21.7	4.3	87.0	4.3
GM1988	43.5	0.0	56.5	17.4	8.7	47.8
Seq13A07	34.8	47.8	0.0	8.7	56.5	17.4
GM2638	30.4	34.8	0.0	13.0	65.2	0.0
IPAHM245	26.1	0.0	39.1	13.0	0.0	13.0

**%A:** allelic contribution of resistant parent (NAMA); **%B:** allelic contribution of susceptible parent (QH243C); **%H:** allelic contribution of hybrids.

## 4 Discussion

The present study was undertaken to identify marker-trait association from a mapping population derived from resistant (NAMA) and susceptible (QH243C) parents for ELS. The populations were phenotyped for ELS followed by genotyping with SSR markers and subsequent identification of potential SSR markers linked to ELS resistance. Results from ANOVA for phenotype data showed highly significant difference among the genotypes for all ELS scoring stages, indicating high genetic variability among the genotypes. It also suggests the two parents are genetically different. Similar variation and transgressive segregation for different stages of leaf spot diseases have been reported earlier in several groundnut lines [29], [30], [31] and for ELS scoring stages [32].

Estimation of genetic variability revealed a magnitude of variation from low to high for PCV and GCV for the three scoring stages of ELS. High values of GCV and PCV were recorded for ELS_II and ELS_III, suggesting the presence of considerable variation among the population. Similar findings of higher values for GCV and PCV of ELS scoring stages were reported [32], [33], [34]. The differences between PCV and GCV estimates were small for all scoring stages suggesting low effect of environment on the expression of ELS.

According to Robinson [35] estimates of heritability in broad sense could range from low (<30%), moderate (30 < H^2^ < 60%) to high (>60%). In the present study, moderate heritability in broad sense was recorded for ELS_I and high for ELS_II and ELS_III, indicating a high response to selection for ELS resistance due to reduced environment influence. These findings are in accordance with previous reports for ELS scoring stages [30], [33], [36], [37]. All scoring stages of ELS exhibited high PCV and GVC coupled with high heritability in broad sense. This result indicates significant role of additive gene action for inheritance of these characters. It also indicates the lesser influence of environment in expression of ELS. Similar results were reported for leaf spot diseases of groundnut including ELS [34], [36].

A total of 103 markers showed polymorphism between the parental lines out of the 179 SSR markers screened across the whole groundnut genome. Thus, 57.54% of markers revealed polymorphism between the parents. This percentage of polymorphism obtained is high compared to those reported earlier [22], [27], [38], [39], [40], which ranged from 6 to 33%. However several studies have also previously reported high percentage of polymorphism comparable to our finding including 70.8–81.0% [23], 52% [25], 99.4% [41], 76.5% [42], and 50% [43] using SSR markers. Being a self-pollinated plant, cultivated groundnut exhibits generally limited polymorphism due to its origin by single hybridization followed by polyploidization [44]. The high percentage of polymorphism (57.54%) obtained in this study, may be due to the distinct nature of the parental lines used.

A cost effective Selective Genotyping approach was employed to analyze the population in the current study instead of genotyping all the plants in the mapping population. Xu et al. [18] discussed the usefulness of selective genotyping for mapping population. Selective genotyping can be used for effective genetic mapping of QTL with relatively small effects as well as for QTL with epistatic interactions or linked QTL. In addition, selective genotyping can be used for fine mapping to narrow down associated genetic regions to less than 1cM or even few candidate genes. In this study 13 SSR markers were identified as linked to early leaf spot disease resistance through SMA. These markers would be of help in linkage mapping and improving resistance to ELS in groundnut. Several studies have previously identified SSR markers associated to leaf spot diseases including ELS [45], [46], [47]. Of the 13 markers identified through SMA, six markers (Seq13E09, GM1883, GM2745, GM1878, TC6E01 and IPAHM509) were earlier mapped associated to ELS resistance [46]. Many of the markers associated with ELS resistance were also earlier identified to be associated to late leaf spot (LLS) disease [29], suggesting that the two diseases could be positively correlated.

Markers accounting for more than 10% of phenotypic variation are considered to be major markers [48]. Several researchers used this approach to establish marker phenotype association where the phenotypes possessed continuous distribution. In the present study, Seq13E09, GM1883, GM2745, GM1878, TC6E01 and IPAHM509 showed percentage of phenotypic variation more than 10%. In selective genotyping analysis, a total of eight SSR markers were identified to be associated to ELS disease resistance. Based on results of selective genotyping analysis, majority of favourable alleles of IPAHM509, AH4-101, GA131 and GM2745 were from susceptible parent (Q243C), suggesting that both parents have favourable alleles affecting ELS resistance. Similar results were reported earlier for leaf spot disease resistance in groundnut [47]; and for drought tolerance in rice [49], [50], [51].

In summary, four markers (GM1911, GM1883, GM1000 and Seq13E09) were identified through both SMA and selective genotyping analysis methods accounting for more than 10% of phenotypic variation. These markers could be used in a marker-assisted selection program of resistant breeding lines for early leaf spot disease.

## Conflicts of interest

The authors declare no conflict of interest.

## Author contributions

AZ conducted the experiment, collected and analyzed the data for his PhD thesis, and wrote the first draft of the paper; PK, YS, MS*r*, MKV, RKV helped AZ in the genotyping of the population and data analysis; MKP mentored and guided AZ in the design of the genotyping approach and association analysis process; MS*a*, PS helped AZ in data analysis, drafting and improving the paper; BRN developed the concept of the experiment; HD mentored AZ during the field work, and edited the final paper.
